# 反复发热、全血细胞减少

**DOI:** 10.3760/cma.j.cn121090-20241129-00492

**Published:** 2025-09

**Authors:** 祎 缪, 菁 张, 涵 张, 重阳 丁, 震 王, 忠兰 苏, 建勇 李, 文瑜 施

**Affiliations:** 1 南京医科大学第一附属医院血液科，淋巴瘤中心，南京 210029 Department of Hematology, Lymphoma Center, the First Affiliated Hospital of Nanjing Medical University, Nanjing 210029, China; 2 苏州大学附属第二医院血液科，苏州 215006 Department of Hematology, the Second Affiliated Hospital of Soochow University, Suzhou 215006, China; 3 南京医科大学第一附属医院核医学科，南京 210029 Department of Nuclear Medicine, the First Affiliated Hospital of Nanjing Medical University, Nanjing 210029, China; 4 南京医科大学第一附属医院病理科，南京 210029 Department of Pathology, the First Affiliated Hospital of Nanjing Medical University, Nanjing 210029, China; 5 南京医科大学第一附属医院皮肤科，南京 210029 Department of Dermatology, the First Affiliated Hospital of Nanjing Medical University, Nanjing 210029, China; 6 南通大学附属医院肿瘤科、血液科，南通 226001 Department of Oncology, Department of Hematology, Affiliated Hospital of Nantong University, Nantong 226001, China

## Abstract

血管内大B细胞淋巴瘤（IVLBCL）是一种罕见类型的大B细胞淋巴瘤。本文报道了一例因“反复发热、全血细胞减少”就诊的病例。患者，女，64岁，既往诊断华氏巨球蛋白血症，曾接受泽布替尼治疗。2023年2月因“反复发热、全血细胞减少”就诊，PET-CT示双侧肾上腺明显肿大，经肾上腺穿刺活检诊断为弥漫大B细胞淋巴瘤，非特指型。患者接受R-CHOP（利妥昔单抗+环磷酰胺+多柔比星+长春新碱+泼尼松）方案化疗，3个疗程后经头颅MRI检查提示淋巴瘤中枢神经系统浸润。回顾病理检查，最终诊断为IVLBCL。尽管积极治疗，但患者疾病持续进展，于2个月后死亡。本文基于多学科层面，结合该病例，从多学科团队协作角度展开论述，旨在为IVLBCL的临床诊疗提供参考意见。

## Abstract (en)

Intravascular large B-cell lymphoma（IVLBCL）is a rare large B-cell lymphoma subtype. We report a patient who presented with “recurrent fever and pancytopenia.” A 64-year-old female patient had previously been diagnosed with Waldenstrom's macroglobulinemia and had received zanubrutinib treatment. In February 2023, the patient revisited due to "recurrent fever and pancytopenia.” A positron emission tomography/computed tomography scan demonstrated significant enlargement of the bilateral adrenal glands. After an adrenal biopsy, she was diagnosed with diffuse large B-cell lymphoma, not otherwise specified. The patient received chemotherapy with the R-CHOP regimen（rituximab + cyclophosphamide + doxorubicin + vincristine + prednisone）. After three treatment courses, a cranial magnetic resonance imaging examination indicated central nervous system infiltration of the lymphoma. After reviewing the pathology of the adrenal biopsy, the final diagnosis was revised as IVLBCL. Despite aggressive treatment, the disease continued to progress, and the patient died two months later. According to a multidisciplinary level, this article discusses the case from the perspective of a multidisciplinary team collaboration, involving imaging, pathology, dermatology, and lymphoma, to provide reference opinions for the clinical diagnosis and treatment of IVLBCL.

## 病历摘要

患者，女，64岁，2020年8月因“乏力、双下肢水肿”就诊于苏州大学附属第二医院。实验室检查示HGB 76 g/L、白蛋白25 g/L、球蛋白40.6 g/L、血清IgM 8.07 g/L；免疫固定电泳发现IgG-κ型和IgM-κ型M蛋白。骨髓细胞形态示浆细胞2％，免疫分型发现15.6％成熟克隆性B淋巴细胞（CD5^-^CD10^-^CD19^+^CD38^±^CD11c^±^CD103^-^FMC7^+^CD23^-^CD200^+^CD79b^+^Kappa^+^Lambda^-^），MYD88 L265P突变检测阳性，CXCR4突变阴性。诊断为华氏巨球蛋白血症（WM），予伊布替尼420 mg，每日1次，治疗效果良好。患者2021年1月因“反复发热”再次就诊，实验室检查示HGB 61 g/L、PLT 74×10^9^/L，进一步评估为疾病进展，更换治疗方案为RCD（利妥昔单抗+环磷酰胺+地塞米松）方案治疗6个疗程。2023年2月患者复查血常规提示全血细胞减少（WBC 1.9×10^9^/L、HGB 70 g/L、PLT 90×10⁹/L），同时伴有发热，体温最高为39 °C，铁蛋白1 120 µg/L。PET-CT示双侧肾上腺明显肿大［最大标准摄取值（SUVmax）23.68］，胆囊壁增厚（SUVmax 17.35），伴有脾肿大（图1）。经肾上腺穿刺活检，结合病理结果，考虑为弥漫大B细胞淋巴瘤，非特指型（DLBCL, NOS），穿刺标本MYD88 L265P突变检测阳性。骨髓穿刺：淋巴细胞比例增高，浆细胞、噬血细胞易见。患者开始接受R-CHOP（利妥昔单抗+环磷酰胺+多柔比星+长春新碱+泼尼松）方案化疗，3个疗程后复查PET-CT显示肾上腺、胆囊壁病变均恢复正常（Deauville评分1分），右侧额叶发现3个结节灶（SUVmax 19.01），进一步的头颅MRI显示脑内多发异常强化灶及出血灶，符合血管内大B细胞淋巴瘤（IVLBCL）中枢神经系统浸润。回顾病理检查，加做CD34染色，确认肾上腺穿刺标本中CD20阳性肿瘤细胞呈血管内浸润表现（图2），最终诊断为IVLBCL。患者随后出现进行性肌力减退、意识障碍，先后予以含有噻替哌、甲氨蝶呤等的方案化疗。然而患者疾病持续进展，2个月后死亡。

**图1:**
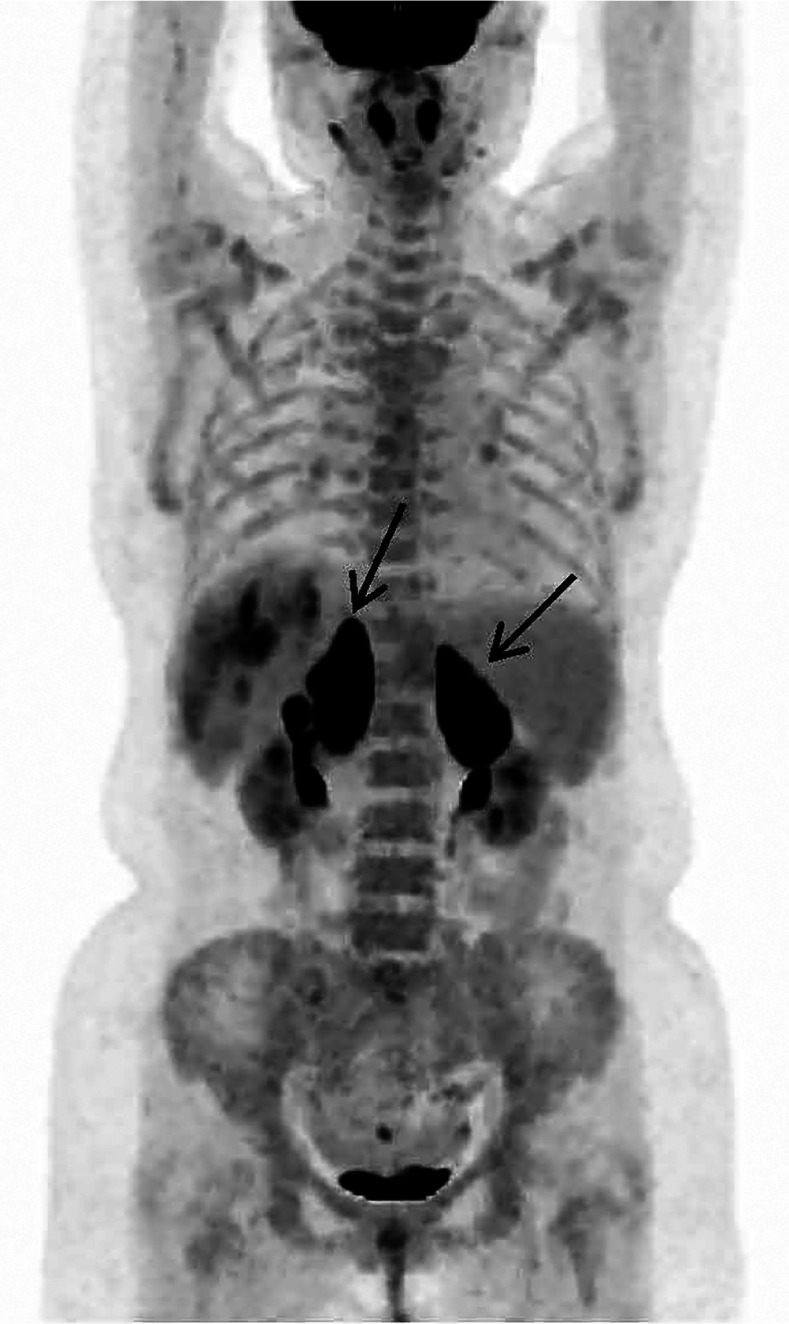
血管内大B细胞淋巴瘤患者2023年2月PET-CT检查结果（箭头所指为双侧肾上腺）

**图2:**
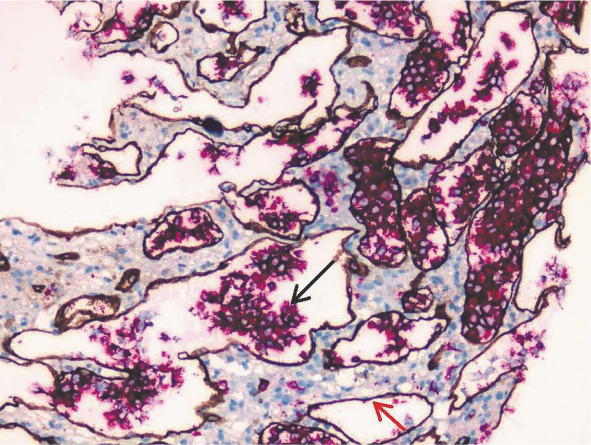
血管内大B细胞淋巴瘤患者肾上腺穿刺活检标本CD20/CD34双染色结果（✕100） **注** 黑色箭头所示红色细胞为血管腔内CD20阳性的肿瘤B细胞；红色箭头所示染色为棕色的CD34阳性的血管内皮细胞

## 临床特征讨论

本例患者为老年女性，2023年2月发病时表现为持续发热伴全血细胞减少、脾肿大，实验室检查示铁蛋白升高，骨髓穿刺示噬血现象。根据HLH-2004标准[1]，本例患者符合噬血细胞性淋巴组织细胞增多症（HLH）诊断。HLH分为原发性和继发性，结合患者年龄，应诊断为继发性HLH。继发性HLH的常见病因为肿瘤、感染及自身免疫性疾病，结合患者B细胞淋巴瘤病史，考虑本例应诊断淋巴瘤相关HLH。但考虑到继发HLH的淋巴瘤绝大部分为侵袭性病理分型，而患者既往诊断为WM，因此需考虑患者WM转为侵袭性淋巴瘤的可能。在老年淋巴瘤相关HLH患者（>60岁）中，大B细胞淋巴瘤为最常见的分型（56.7％）[2]。根据日本的一项研究[3]，在大B细胞淋巴瘤相关HLH患者中，DLBCL，NOS和IVLBCL为最常见的病理分型。因此，在本例患者中，从临床特征角度，需要考虑诊断为DLBCL，NOS或IVLBCL。

## 影像学讨论

本例患者2023年2月PET-CT影像学表现为两侧肾上腺明显肿大伴^18^F-氟代脱氧葡萄糖（^18^F-FDG）代谢增高。结合受累部位以及SUVmax，首先考虑侵袭性淋巴瘤肾上腺累及。对于影像学上表现为双侧肾上腺受累的侵袭性淋巴瘤，主要的病理分型为大B细胞淋巴瘤，在大B细胞淋巴瘤中，除DLBCL，NOS外，还包括IVLBCL和EB病毒阳性DLBCL。患者最终诊断为IVLBCL，其影像学特征亦支持。

IVLBCL在不同的影像学检查中可能表现不同。在普通的平扫CT上，约20％的病例在肺窗上表现为磨玻璃影，脾肿大常见（85％）[4]。对于怀疑IVLBCL或确诊IVLBCL的患者，推荐行PET-CT及中枢神经系统MRI检查。并非所有IVLBCL患者在PET-CT影像学上均有表现，部分患者PET-CT上无^18^F-FDG高代谢病灶[5]。在PET-CT影像上，部分患者可表现出双肺^18^F-FDG代谢呈轻中度不均匀弥漫性增高[4]；部分患者可见肝脾肿大伴^18^F-FDG弥漫代谢增高，部分患者可见肝脾内^18^F-FDG代谢明显增高结节、肿块影；肾脏累及的患者可表现为肾脏弥漫性肿大，^18^F-FDG代谢弥漫性增高[4]。IVLBCL的肾上腺累及常见，PET-CT上可表现为肾上腺肿块^18^F-FDG代谢增高[4],[6]。PET-CT影像学上IVLBCL皮肤受累表现少见[7]，可表现为皮下脂肪内絮状低密度影伴^18^F-FDG代谢轻度增高。约70％的IVLBCL病例骨髓^18^F-FDG代谢增高，其中1/3的患者表现为局限性^18^F-FDG代谢增高，另外2/3的患者表现为弥漫性^18^F-FDG代谢增高[4]。根据Matsue等[4]的研究，约26％的IVLBCL会出现淋巴结^18^F-FDG代谢增高。脑部MRI异常在IVLBCL患者中常见（86.2％），其中最常见的异常包括T2加权成像高信号异常（54.1％），其他异常包括非特异性脑白质病灶（45.9％）、梗死样病灶（27.0％）和脑膜强化（10.8％）[4]。本例患者后期出现中枢神经系统累及，其脑部MRI表现为T2加权成像高信号异常，符合IVLBCL的脑部MRI特征。

## 病理诊断及病理特征讨论

IVLBCL的诊断依赖于病理活检。对于有明确受累部位的患者，在保障穿刺安全的前提下，推荐行相应部位的活检，如对于皮肤受累的IVLBCL，推荐累及部位的皮肤活检，此外骨髓、肾上腺、肺、肾、肝也是常见的活检部位[8]。本例患者PET-CT影像学提示肾上腺受累，且病灶较大，PLT及凝血功能符合穿刺条件，因此肾上腺穿刺活检安全性可，成功率较高。单纯的骨髓活检仅能诊断约20％的IVLBCL患者，主要因为仅有60％左右的IVLBCL患者存在骨髓累及，且在伴骨髓累及的患者中仅1/3表现为单纯的血窦内侵犯[4]。

此外，对于疑似IVLBCL但未明确病灶或相关病灶穿刺风险较大的患者，随机皮肤活检（RSB）是最为重要且有效的诊断手段。根据一项来自日本的大样本回顾性研究[9]，RSB诊断IVLBCL的敏感性为77.8％、特异性为98.7％。RSB的阳性预测值为96.6％、阴性预测值为90.6％。此外，该研究[9]还发现了RSB阳性的预测因素，包括：不明原因发热、意识改变、低氧血症、PLT<120×10^9^/L、LDH>800 U/L以及血清可溶性白细胞介素2受体（sIL-2R）水平>5 000 U/ml。如具有5个及以上的预测因素，则RSB阳性概率高达65％；如仅有2项及以下的预测因素，则RSB阳性概率为0。因此，这些预测因素对于决定是否进行RSB具有重要的指导意义。RSB的诊断准确性取决于活检的位置、数量、深度和宽度[10]。在西方国家环钻活检常被用于诊断IVLBCL，但环钻活检标本中缺少足量的皮下脂肪组织[10]。由于大多数IVLBCL病变位于皮下脂肪组织，且病变发生在真皮层的频率较低，RSB标本应包含足够数量的皮下脂肪组织以供诊断。因此，建议切口活检深度距离皮肤表面超过5 mm，取材部位为大腿两侧及腹部等三处甚至更多部位采集。《血管内大B细胞淋巴瘤诊治中国专家共识（2023年版）》[11]推荐RSB必须深达肌层，虽然部分病例可能并不一定需要深达肌层，但必须强调的是RSB达到足够的深度对于采集到阳性病灶至关重要。

从病理学上讲，IVLBCL是一种以肿瘤性大B细胞局限于血管腔内增殖为特征的侵袭性结外B细胞淋巴瘤。IVLBCL可以在小血管腔内，尤其是毛细血管内，观察到具有泡状核和单个或多个明显核仁的大淋巴细胞，通常伴有丝分裂象；可以观察到不黏附、黏附或向血管外迁移/黏附的生长模式。本病例肾上腺穿刺病理中表现为典型的血管内肿瘤细胞浸润模式。在极少数情况下，淋巴瘤细胞可表现出间变样形态。在噬血细胞变异型中，可以见到不同程度的非肿瘤性组织细胞浸润，并伴有噬血现象[4]。免疫表型方面，肿瘤细胞强表达CD20。约13％的IVLBCL病例表达CD10，75％～80％的病例表达MUM1且具有非生发中心B细胞免疫表型。除此之外，22％～52％的IVLBCL表达CD5[8],[12]，44％表达PD-L1[13]。分子遗传学方面，常见基因突变：MYD88突变（57％）、CD79B突变（67％）、SETD1B突变（57％）和HLA-B突变（57％）[14]。IVLBCL中具有CD274（PD-L1）和PDCD1LG2（PD-L2）基因的结构性异常[14]。现有的研究表明，在检测分子遗传学异常方面，循环肿瘤DNA（ctDNA）与来自肿瘤DNA相比更具有优势。

## 治疗讨论

IVLBCL的治疗包括支持治疗和疾病治疗两个方面。部分IVLBCL患者诊断时常伴HLH，因此，在部分患者中，需要ICU团队的参与，给予机械通气、连续肾脏替代疗法（CRRT）及血浆置换等高级生命支持治疗。推荐在以上有效的生命支持治疗的保障下尽快针对本病进行化疗及靶向治疗。

目前对于IVLBCL本病治疗选择，尚缺乏随机对照研究。既往回顾性研究[15]表明，含有利妥昔单抗的免疫化疗方案相比于单纯的化疗方案显著改善了IVLBCL患者的预后。对于初治时伴有HLH的患者，可采用HLH-94方案或DEP方案（优先推荐）控制HLH，待HLH及一般状况改善后再针对淋巴瘤进行治疗。IVLBCL具有很高的中枢神经系统复发率（10％～25％）[16]。PRIMEUR-IVL研究[17]是一项多中心、单臂、Ⅱ期临床试验，也是针对IVLBCL的首个前瞻性临床试验，该研究评估R-CHOP方案联合大剂量甲氨蝶呤（HD-MTX）和鞘内注射对初诊且不伴中枢神经系统受累IVLBCL患者的疗效和安全性，中位随访3.9年，2年无进展生存（PFS）率和总生存（OS）率分别为76％和92％，2年内中枢神经系统复发率为3％。因此，R-CHOP方案联合HD-MTX可作为初诊且无中枢神经系统受累IVLBCL患者的一线治疗方案。但目前静脉用HD-MTX预防复发仍具挑战，根据Terao等[16]的研究，在51例接受HD-MTX预防的IVLBCL患者中枢神经系统复发率仍达9.8％。需要指出的是，PRIMEUR-IVL研究中伴有HLH患者的比例较低，因此合并HLH的IVLBCL的一线方案仍需探索。

IVLBCL中MCD亚型比例较高，因此联合布鲁顿酪氨酸激酶抑制剂可能获益[14]。北京协和医院一项前瞻性、单臂、Ⅱ期临床试验评估泽布替尼联合R-CHOP（ZR-CHOP）方案治疗初诊IVLBCL患者的疗效和安全性，入组患者接受8个疗程ZR-CHOP方案的治疗，截至2023年2月，研究共入组23例患者，其中13例完成8个疗程后12例取得完全缓解、1例部分缓解。中位随访439 d，完成8个疗程的患者无复发[18]。此外，该方案安全性可控。综上，ZR-CHOP方案治疗IVLBCL较有前景。一线治疗缓解后序贯auto-HSCT的IVLBCL患者3年PFS率和OS率分别为83％和89％，3年累积复发率为14％，提示巩固治疗可能带来获益[19]。

难治复发的IVLBCL患者预后较差。对于复发的患者，可在挽救方案治疗有效后序贯auto-HSCT巩固。可根据IVLBCL中枢神经系统是否复发选取不同的挽救治疗方案。亦有文献报道CAR-T细胞治疗在复发IVLBCL患者中的应用[20]，但其在难治复发IVLBCL中的疗效仍有待验证。

本病例体现出了IVLBCL病理诊断的重要性，尽管IVLBCL属于大B细胞淋巴瘤，但其诊疗方案与一般的DLBCL，NOS并不完全相同。在IVLBCL的诊疗过程中，基线中枢神经系统受累状况的评估以及中枢神经系统预防非常关键。IVLBCL是一种具有挑战性的疾病，需要包括淋巴瘤科、影像科、核医学科、病理科以及皮肤科在内的多学科合作以优化诊疗策略。通过多学科的综合诊疗，IVLBCL患者能够得到及时的诊疗，部分能够保持长期缓解。对于诊断疑难的患者，ctDNA可以起到辅助诊断的作用。对于难治复发的患者，挽救性治疗方案和auto-HSCT可能是有效的治疗选择。未来需要更多研究来探索IVLBCL的最佳治疗方案。
